# Exploring the relationships between first impressions and MMI ratings: a pilot study

**DOI:** 10.1007/s10459-022-10151-5

**Published:** 2022-09-02

**Authors:** Dietrich Klusmann, Mirjana Knorr, Wolfgang Hampe

**Affiliations:** Institute of Biochemistry and Molecular Cell Biology, University Medical Center Hamburg-Eppendorf (UKE), N41, Martinistr, 52, 20246 Hamburg, Germany

**Keywords:** First impression, MMI, OSCE, Rater-based assessment, Rater cognition

## Abstract

The phenomenon of first impression is well researched in social psychology, but less so in the study of OSCEs and the multiple mini interview (MMI). To explore its bearing on the MMI method we included a rating of first impression in the MMI for student selection executed 2012 at the University Medical Center Hamburg-Eppendorf, Germany (196 applicants, 26 pairs of raters) and analyzed how it was related to MMI performance ratings made by (a) the same rater, and (b) a different rater. First impression was assessed immediately after an applicant entered the test room. Each MMI-task took 5 min and was rated subsequently. Internal consistency was α = .71 for first impression and α = .69 for MMI performance. First impression and MMI performance correlated by r = .49. Both measures weakly predicted performance in two OSCEs for communication skills, assessed 18 months later. MMI performance did not increment prediction above the contribution of first impression and vice versa. Prediction was independent of whether or not the rater who rated first impression also rated MMI performance. The correlation between first impression and MMI-performance is in line with the results of corresponding social psychological studies, showing that judgements based on minimal information moderately predict behavioral measures. It is also in accordance with the notion that raters often blend their specific assessment task outlined in MMI-instructions with the self-imposed question of whether a candidate would fit the role of a medical doctor.

## Introduction

The Multiple Mini Interview (MMI) format is widely used in the selection of medical students. It typically entails a series of short tasks designed to assess facets of social competence such as communication skills and empathy in a role play (Eva et al., 2004; Knorr & Hissbach, 2014). Assessment is completed after a short time span, typically 5–10 min, raters are not supposed to be acquainted with the candidates, rating guides and training are provided, but the step from observations to ratings on abstract dimensions allows for much leeway.

This ambiguity opens room for spontaneous inference from appearance which may sway judgement one way or the other. So, the question arises how first impressions might influence the rating process. A search for research examining the importance of first impression for MMI- and OSCE-type measures revealed only four studies (Christensen et al., 2018; Gingerich et al., 2017; Wood et al., 2017, 2018). This dearth of interest is astonishing, since the topic of first impression abounds in the social psychology literature and has been researched for decades at an increasing level of sophistication (Ambady & Skowronski, 2008; Perret, 2012; Todorov, 2017) and a research program for the role of first impression in rater based assessments has been proposed Wood (2014).

In a conceptual analysis of workplace-based assessments, Gingerich et al. (2014) come to the following conclusion: “There appear to be three distinct, although not mutually exclusive, perspectives on assessor cognition within the research community. The first perspective describes potentially controllable cognitive processes invoked during assessment and draws on components of behavioural learning theory to help frame an approach to reduce unwanted variability in assessors’ assessments through faculty training. The second perspective draws on social psychology research and focuses on identifying the automatic and unavoidable biases of human cognition so that assessment systems can compensate for them. A third perspective draws from socio-cultural theory and the expertise literature and proposes that variability in judgements could provide useful assessment information within a radically different assessment design” (p. 1056). This article draws much from social psychological research on cognitive bias, but the topic of first impression or judgement based on minimal information is not mentioned. First impression is automatic and subject to biases of human cognition (second perspective), and it is a process of making sense of complex scenarios through inference and contextual sensitivity (third perspective). However, it is neither simply an expression of cognitive bias nor completely idiosyncratic. First impression, as investigated in social psychological studies, does not unpredictably vary from person to person, but is uniform and informative to some extent, as attested by high rater agreement and moderate correlations with more objective measures of outcome (Todorov, 2017). This may also be true for first impression in MMI-like assessment–situations. If so, first impression will form a fairly consistent scale which correlates with more objective measures, in this case MMI-performance and OSCE-scores.

To evaluate this conjecture, we analyzed a rating of first impression with regard to its internal consistency as a scale, its correlation with a subsequent MMI-rating, and its performance as a predictor for OSCE-scores obtained 18 month later. As we are convinced that the body of knowledge about first impressions gathered in social psychological experiments can help to clarify MMI-type measurement—to open the black box so to speak (Gingerich et al., 2014), we shall start with a summary of research on person perception under the condition of minimal information.

### Zero acquaintance

In the person-perception literature the terms "first impression", "thin slices of expressive behavior", and "zero acquaintance" are often used interchangeably. The first studies in the late 1960s showed surprising accuracy of judgements based on minimal information about strangers (Kenny & West, 2008). In typical experiments, subjects responded to still photographs, samples of recorded voice, video clips, and face-to-face situations without conversation. Even when this information was seemingly unrelated to the assessment task, often a judgement of personality traits, consensus amongst subjects was high and measures corresponded with self-ratings and third party assessments (Zebrowitz & Montepare, 2008). First impressions of extraversion and conscientiousness correlated with self-assessments of the same traits in a range of 0.25–0.65 (Gray, 2008).

In a meta-analysis, Ambady and Rosenthal (1992) determined how accurately short observations by strangers predicted behavioral outcomes such as supervisor evaluation, teacher effectiveness, patient compliance, and deception. The overall effect size was r = 0.39, equivalent to d = 0.85. Neither length of exposure nor channel of information (face, speech, body movement, voice) was related to accuracy of prediction. The authors concluded that perceivers are typically able to predict behavior from thin slices of information. They suggested three tentative explanations: (1) An automatic brain system for the rapid evaluation of other persons evolved in evolutionary history. (2) Stereotypes activated by minimal cues derived from first impressions may improve guesses in the way priors are used in Bayesian prediction. (3) Snap judgement is often focused on cues that really matter, whereas explicit deliberation is easily distracted by irrelevant detail. This has been shown in many experiments, e.g. as reviewed in Wood (2014).

### The face

Since the face is a prominent source of first impressions it has been investigated most thoroughly to reveal the specific features that lead to rater agreement. Impressions from faces are literally single glance impressions—they do not change much with presentations longer than one fifth of a second (Todorov et al., 2008). Interpersonal agreement on first impressions of faces is generally high, but correlations with more objective trait measures are lower.

Todorov et al. (2008) found trait descriptions based on facial appearance to be well represented in a space defined by two dimensions: trustworthiness and dominance. Trustworthiness (or valence) concerns the perceived intention to help or to harm. Dominance (or power) concerns the perceived capability to carry out any such intentions. Equivalent dimensions are found consistently in more general research on person perception, mostly called "warmth" and "competence" instead of "trustworthiness" and "dominance". As these dimensions help in appraising whether a person will become a threat or a supporting partner (Sutherland et al., 2016), their salience has probably arisen as an evolutionary adaptation to human group life (Schaller, 2008). Experiments with noise-distorted images show that faces suggesting traits such as competence or trustworthiness, have distinctive features, differentiating them from faces suggesting a lack of these traits (Todorov, 2017).

The high agreement between observers of faces seems to reflect a uniformity of the mental processes the human mind uses to arrive at instantaneous evaluations of facial features. Neuroimaging studies point to circumscribed brain systems specialized for this task (Rule & Ambady, 2008; Schiller et al., 2009). Neural response patterns in the fusiform gyrus and related areas predict associated cognitive states such as stereotype representations of gender, race, identity and emotion. Actual face perception is influenced by these category representations (Brooks et al., 2021).

### Halo effect

Global impressions can influence the way more specific attributes are evaluated which is known as the halo effect. It has true and illusory components—valid halo and invalid halo. A halo effect may inflate correlations among ratings, magnify differences in the mean ratings of different individuals and flatten the overall profile of ratings. However, a halo effect does not always diminish the validity of specific measures—it may enhance validity whenever the overall halo-evaluation provides a valid preview of the subsequent more specific evaluation (Murphy et al., 1993). # Wood (2014) Therefore, controlling halo sometimes will do more harm than good (Murphy et al., 1993).

### Attractiveness

Attractive people are perceived as particularly likable, outgoing, socially competent, powerful, intelligent, and healthy, and these qualities are in fact often found to be associated with attractiveness, albeit to a much smaller degree than the high consensus among raters suggests (Zebrowitz & Montepare, 2008). Sometimes the attractiveness-halo is misleading. In a study by Talamas et al. (2016), standardized face images of university students were rated for attractiveness, expected academic performance, intelligence, and conscientiousness. The attractiveness rating correlated highly with the aforementioned ratings, but not with actual academic performance. Only perceived conscientiousness correlated with actual academic performance and this correlation increased after the effect of perceived attractiveness was partialled out. This points to perceived attractiveness as a confounder for an assessment that would otherwise be more accurate.

### Accuracy of first impression

Accuracy of first impression (AFI) in one domain is not a good predictor of AFI in another, therefore AFI cannot be considered a unitary skill (Hall & Andrzejewski, 2008). AFI is only weakly correlated with general intelligence, and positively associated with social status, mental health, and social adjustment (Gray, 2008). The ability to detect personality characteristics from the face depends on the personality of the judge. Depressed people are particularly sensitive to phoniness, extraverts are especially good at identifying their own kind (Gray, 2008). People with Machiavellian traits (interest in manipulating people, cold-hearted) are good judges of faces. In contrast, warm-hearted and generous people seem to be poor judges of faces, perhaps because, as Perret writes: "Their greater compassion means that they treat all people alike “ (Perret, 2012, p. 185).

### Ability to generate a favourable first impression

In a study of first impression based on brief video clips of self-introduction (Back et al., 2010) narcissists were judged particularly favorable by unacquainted observers. This positive impression was mediated by neat appearances, charming facial expressions, self-assured body movements, and humorous verbal expressions. It prevailed even when sound was turned off. Narcissim was self-assessed by a personality inventory with 4 subscales: leadership/authority, self-absorption/admiration, superiority/arrogance, and exploitativeness/ sense of entitlement. Of these subscales, the tendency to exploit others and to feel entitled to some preference was most closely related to a favorable impression at first sight, thus manipulative narcissists seem to be able to make others like them—at least in the short run.

### OSCE-like methods and first impression

We found only four studies investigating first impression in OSCE-like methods. In two of them, the term first impression was used explicitly (Wood et al., 2017, 2018); in two others first impression was not mentioned, but concepts such as "taste" and "mental shortcuts" were used which are related to first impression (Christensen et al., 2018; Gingerich et al., 2017).

Wood et al. (2017) scripted a history-taking OSCE and reenacted it 6 times with professional actors playing the role of examinees. The videotapes of these reenactments were shown to 23 raters who recorded their first impression and subsequently rated the OSCE-performance. First impressions correlated moderately with later OSCE-performance. A similar study from the same lab by Wood et al. (2018) investigated the willingness of raters to modify first impressions in the face of improvement vs. deterioration during an OSCE-trial. Such changes were in fact taken into account and final ratings differed from first impressions accordingly.

Of the two studies which did not mention the concept of first impression, the first is a qualitative study based on 12 post hoc intensive interviews with raters. It centers on the concept of taste, conceived as a frame of tacit categories, used to distinguish between good and bad performances (Christensen et al., 2018). Raters differed in their evaluation of qualities like rapport building, emotional resonance, tactfulness, medical expertise, resilience, and rationality, depending on their enculturation in different institutional cultures, be it medical, or psychological. Like connaisseurs of art or food, different raters emphasized different criteria in their evaluations. According to the authors such differences should not be treated simply as nuisance variance—they reflect a variety of different ways to judge social competence in clinical work life which may improve assessment when taken into account in their full range. This corresponds to the third of three ways to conceive of assessor variability in Gingerich et al (2014), the assessor as meaningfully idiosyncratic: "experts are capable of making sense of highly complex and nuanced scenarios through inference and contextual sensitivity, which suggests assessor differences may represent legitimate experience-based interpretations”, p.1055). The other two ways were concerned with trainability and fallibility.

Tavares and Eva (2013) criticize that rater-based assessments in health professional education have "generally been implemented with limited consideration of human cognitive and perceptual limitations" (p. 291). Assessing multiple aspects of behavior generates a mental workload that cannot be sustained indefinitely. As a result, observations, obvious to the unburdened observer, may go unnoticed (inattentional blindness). The authors do not delve deeper into a specification of the shortcuts to which raters facing cognitive overload may resort—this should have led naturally to the heuristics underlying first impression.

To summarize research on first impressions: Agreement between raters based on first impressions is generally high and exceeds factual validity. First impressions may be surprisingly accurate, depending on stimulus, context, and task; they may also be misleading, especially when distracting from more reliable cues.

## The present study

Since 2009 the University Medical Center Hamburg-Eppendorf employs an MMI for student selection (Hissbach et al., 2014; Knorr et al., 2018). At the occasion of the testing 2012 we added a rating of first impression to the MMI-procedure to investigate these three questions:

1. Is the first impression rating consistent across raters and stations?
2. How is first impression related to MMI performance?
3. How do first impression and MMI performance rating combine in the prediction of social competence measured by communication skill OSCEs 18 months later?

For each of these questions we will examine how results differ if ratings for first impression and MMI performance are obtained from the same rater, as compared to different raters.

## Methods

In 2012, the MMI for medical student selection in Hamburg was the last step of an admission procedure (Hissbach et al., 2011) based on scores in educational attainment and natural science (Meyer et al., 2019). The MMI was attended by 196 applicants, of which 111 consequently were admitted to study. It was designed to measure social competency with a series of 8 stations:

1. Role play: Decline a patient’s demand for excessive massage prescriptions.
2. Interview: Identify emotional messages that might be hidden in short statements.
3. Role play: Confront a teacher about a complaint, speaking for the class.
4. Interview: Describe the doctor-patient relationship shown in a short video clip.
5. Interview: Discover possible meanings in an emotionally ambiguous utterance.
6. Interview: Describe character traits you don’t like in people.
7. Interview: Evaluate different ways a husband may respond to the mastectomy of his wife.
8. Role play: Explore why a coworker seemingly avoids to join a company excursion.

Even when some stations played out in a medical context, the ratings were not intended to reflect medical knowledge. Raters were recruited from the clinic’s medical and psychological staff and instructed one day before the test in an afternoon session of 4 h, including trial runs with students acting as applicants. For each station, behavioral anchors for the 5 categories of a global performance rating were provided.

The MMI had four parallel circuits with three role play and five interview stations designed to assess social competence amounting to a total of 8 × 4 = 32 stations. In 26 of these 32 stations two raters were present in each room. This enabled us to compare the assessments of a rater 1 and a rater 2. Of the 2 * 26 = 52 raters in doubly manned stations 28 were female and 24 were male. Raters were either physicians (n = 28), psychologists (n = 20), or faculty employees with another academic background (n = 4). Within rater-pairs the profession was balanced as much as possible. Rater leniency did not depend on gender or profession (no main effect). Overall mean ratings in the five-point rating scales were M = 3.64, SD = 0.85 for first impression and M = 3.48, SD = 0.98 for the MMI. Some differences between subgroups composed by the intersection of gender and profession were found, e.g. female physicians were less lenient than female psychologists, but all these differences were small (Knorr et al., 2014).

Applicants had 90 s to read the instructions at the door of the examination room. After a signal they entered the room and started a five-minute interaction with an interviewer or a simulated patient. The two raters also present in this room were instructed to record their first impressions within 30 s after the entrance of the applicant and in any case before the start of the MMI-task. Within this time span, raters had the chance to observe how the person entered the room, greeted the interviewer, took a seat, the physiognomy of the face, changes in facial expressions, directions of gaze, dress, hairstyle, body movements, sound of voice, the way rapport with the interviewer was established, and many more features of appearance. First impression (FIR) was rated on a 5-point scale in response to the question: "Does this applicant appear to be qualified for studying medicine?". Subsequently the MMI task commenced and after 5 minute performance was rated summarily on a 5-point scale (MMI).

This design ideally would yield a data matrix with 8 × 4 = 32 pairs of raters nested under 8 stations and 4 floors (Table 1). However at 8 of 32 stations the second rater was missing.

**Table 1:**
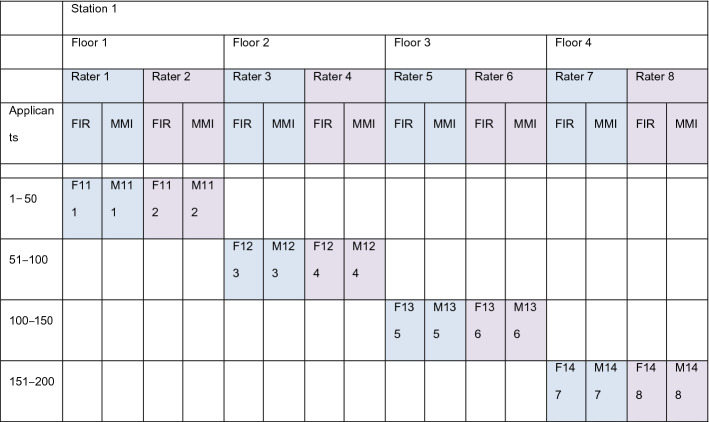
Data matrix for one of 8 stations: raters are nested under floors and under applicants

Each rater encounters 48 of 192 applicants and each rater makes two assessments: first impression (FIR) at the start, and MMI-performance (MMI) at the end of each encounter. With 4 pairs of raters for each station there are 2^4^ = 16 permutations of data vectors with at least one exchange of who is designated rater_1 and rater_2. This data structure is repeated for 8 stations, yielding 2^8^ = 256 permutations, not all of them realized, because at 8 of 32 stations the second rater was missing

Correlations between rater 1 and rater 2 are unambiguously defined only at the level of a single station, but not at the level of the total sample, because pairs of raters changed from station to station and it was arbitrary who should be coded 1 or 2. Therefore, the correlation in the total sample may change slightly depending on which code designations of rater 1 and rater 2 have been swapped at some station. If at every station two raters were present, with 8 stations 2^8^ = 256 permutated datasets were possible with rater-designations swapped at least once (Table 1). We did not calculate correlation coefficients for all these permutations but stopped at 8 repeated calculations because the dispersion of the correlation coefficients due to permutations was negligible. The correlation between rater 1 and rater 2 in the total sample was computed as the mean correlation of these 8 permutated repetitions. All coefficients were arctanh-transformed before averaging, then inversely transformed.

At each station, the mean of the two raters was taken as the applicant’s score for first impression (FIR) and MMI performance (MMI), respectively. The total score was the mean of this score over all stations. This is also the score which had been used for the admission decision (in combination with additional information). We also computed individual scores for rater 1 and rater 2 (single-rater-scores) for FIR and MMI. Correlations between FIR and MMI were computed in two ways:

1. Mean correlations: For each individual station a correlation between FIR and MMI was computed. Then these 8 station-specific correlations were averaged.
2. Correlations of means: Total means for FIR and MMI across all 8 stations were computed. Then these two means were correlated.

Internal consistencies were evaluated across stations using Cronbach’s α and a reliability coefficient obtained from a nominal Item Response Theory model (Bock, 1972; Thissen et al., 2010), the marginal reliability of response pattern scores.

In a further step we used a provisional criterion for predictive validity: the mean performance at two OSCE-stations taken 18 months after admission, one for assessing communication skills and the other for taking a case history. The relative contributions to predictive validity of first impression and MMI performance were evaluated by multiple regression analysis using mean OSCE-ratings as the criterion and three definitions of predictors: (1) scores for first impression and MMI averaged across two raters, (2) scores for first impression and MMI made by the same rater, (3) scores for first impression and MMI made by different raters. By comparing definitions 2 and 3, the effect of within-rater dependency on prediction can be assessed.

Statistical software was SPSS 25 and IRTPRO 4.0.

## Results

### Consistency of first impression ratings

With two raters and two dimensions, 6 correlations within each station can be calculated. Mean correlations across all 8 stations (mean of correlations, Table 2, upper triangle) show that (1) agreement between raters was lower for first impression than for the MMI performance rating, and (2) correlations between first impression and MMI performance ratings were slightly higher when both ratings were made by the same rater, as compared to different raters. When the station-specific ratings were averaged across stations to yield a total score, a single correlation could be computed for each variable (correlation of means, Table 2, lower triangle). With this method of computation nearly the same correlational pattern emerged, albeit with higher values.

**Table 2:** Correlations between first impression and MMI-rating as assessed by rater 1 (FIR_1_, MMI_1_) with first impression and MMI-rating as assessed by rater 2 (FIR_2_, MMI_2_)

	FIR_1_	FIR_2_	MMI_1_	MMI_2_
FIR_1_	1.00	**.34**	**.28**	**.21**
FIR_2_	*.66*	1.00	**.21**	**.34**
MMI_1_	*.46*	*.38*	1.00	**.56**
MMI_2_	*.37*	*.43*	*.75*	1.00

Upper diagonal: Mean of correlations (correlations of ratings were computed separately for 8 stations, then averaged)

Lower diagonal: Correlation of means (means of ratings across 8 stations were computed, then correlated)

Internal consistency across stations amounted to Cronbach’s α = 0.71 for first impression and α = 0.69 for MMI performance. As explained in the methods section, these coefficients are mean values obtained after permutating the designations of who was rater 1 and rater 2 eight times. The marginal reliability of response pattern scores obtained from a nominal IRT model was 0.69 for first impression and 0.65 for MMI performance. Thus, both methods resulted in a moderate level of internal consistency across stations for both measures. This is also revealed in similar item response characteristics of the station-specific ratings for first impression and MMI-performance. When data from single raters were used instead of the mean of two raters, internal consistency was α = 0.57 for first impression and α = 0.57 for MMI performance.

### Correlation of first impression with MMI performance

**Fig. 1:**
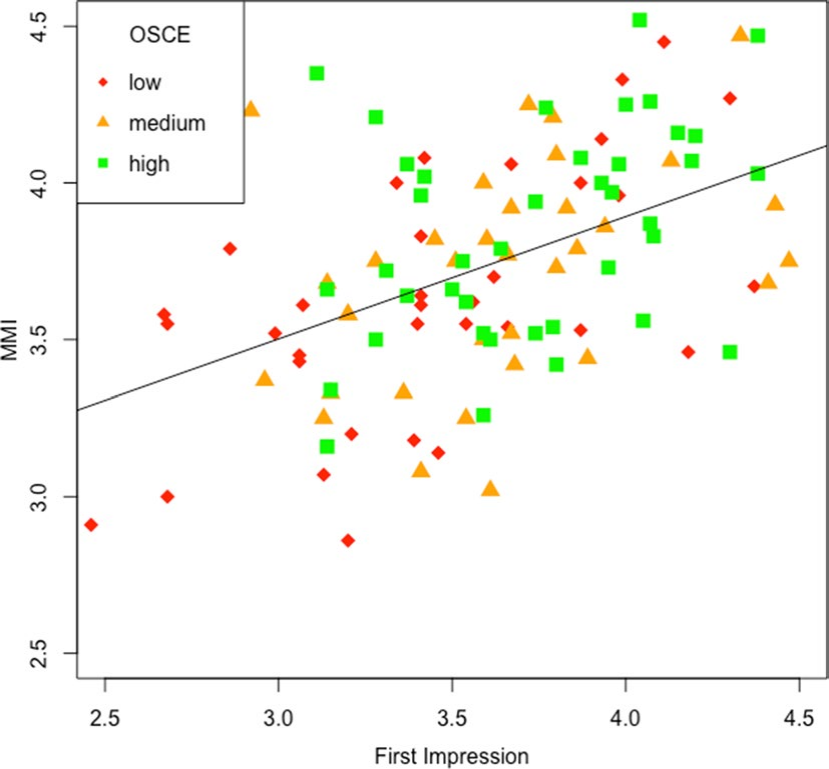
Scattergram of first impression with MMI-performance rating, for 3 subgroups with low, medium, and high performance in two psychosocial OSCE stations assessed 18 months after the entrance test involving the MMI. Sample: 111 applicants of the cohort of applicants, 2012 who were admitted to study, correlation: r = .49

The correlation between the total scores for first impression and MMI-performance, both taken as the mean of two raters across all 8 stations, was r = 0.49 in the total sample of all 192 applicants, and yielded the same value in the subsample of applicants admitted to medical school (n = 111). The scattergram for this subsample is shown in Fig. 1. If only a single rater was used to compute the scores there were two possibilities: (1) the same rater rated first impression as well as MMI-performance, (2) ratings were made by different raters. In the same-rater-condition, the mean correlation computed across stations (with permutations of who was designated rater 1 and rater 2) was r = 0.44. In the different-raters-condition it was r = 0.37.

### Prediction of psychosocial OSCEs by first impression and MMI performance rating

The mean performance at two OSCE-stations taken 18 months after admission is not a measure of high psychometric dependability, nevertheless it allows to assess validity at least provisionally. Figure 1 shows the relations between first impression, MMI, and OSCE-performance. In the lower left part of the scattergram where both, first impression-ratings and MMI-ratings, are low, OSCE-performance tends to be low (red) whereas in the center and the upper right part medium and high performance (orange and green) prevails. Thus, both measures, first impression and MMI, look like weak predictors of OSCE-performance and seem to predict low performance better than high performance.

A regression model (Table 3, model 3) with OSCE-performance as the criterion yielded a significant prediction effect (p(F) = 0.009) of small size (8.4% explained variance). MMI performance did not significantly increment validity above the contribution of first impression (p(F) = 0.076) and vice versa (p(F) = 0.142). With two predictors, explained variance is composed of two unique contributions and one joint contribution: The unique contributions were 1.9% for first impression and 2.7% for MMI performance, the joint contribution of both predictors was 3.8%. Thus, for nearly one half of the explained variance, the predictive effect of first impression is indistinguishable from the predictive effect of MMI-performance. Removing the variance due to first impression from the MMI performance rating by computing the residuum lowered the correlation with psychosocial OSCE performance from a first order r = 0.255 to a semi-partial r = 0.165.

**Table 3:** Prediction of the mean performance in two psychosocial OSCE stations (OSCE) by first impression (FIR) and MMI performance (MMI)

	R^2^	P(F)	Beta	Sig. (beta)	First-order corr	Semi-partial corr	R^2^ change	Sig. (R^2^ change)
Model 1 First Impression only	.057	.012*						
FIR			.238	.012*	.238			
Model 2 MMI performance only	.065	.007**						
MMI			.255	.007** *	.255			
Model 3 FIR and MMI	.084	.009**						
FIR			.153	*.142*	.238	.136	.019	*.142*
MMI			.185	*.076*	.255	.165	.027	*.076*

The predictors FIR and MMI are mean ratings across two raters. The coefficients in this table are representative of 8 multiple regression runs obtained with different permutations of designations as rater 1 or rater 2. The differences between these runs are small and do not change any conclusion from the data. If instead of means across two raters scores from a single rater are used as predictors explained variance drops by a small amount but the pattern of coefficients stays the same

The semipartial correlation is the correlation of residuals with the OSCE-criterion where the effect of MMI is taken out of FIR and vice versa

Prediction did not drop off much when data from a single rater was used instead of the mean of two raters. For a single-rater-measure, two cases have been distinguished: (1) first impression and MMI performance were rated by the same rater, and (2) first impression and MMI performance were rated by different raters. The two cases yielded similar results in multiple regression analysis.

## Discussion

Main findings were:

1. The internal consistency of first impression was moderate (α = 0.71) and about equal to the internal consistency of MMI performance.
2. The total scores for first impression and MMI performance correlated with r = 0.49
3. Both measures weakly predicted the mean of two psychosocial OSCEs obtained 18 months later.
4. MMI performance did not significantly increment prediction above the contribution of first impression and vice versa.
5. Prediction did not depend on whether the rater who rated first impression also rated MMI performance or MMI performance was rated by a different rater.

### Internal consistency of first impression

Rater agreement in first impression was low when computed as the mean of 8 station-specific correlations (r = 0.34) and higher (r = 0.66) when measured as the correlation of mean scores obtained by averaging over all 8 stations, separately computed for rater 1 and rater 2 ( allowing for permutations, as explained in the methods section (Table 1)). The contrast between mean of correlations and correlation of means probably arises because each single station-specific correlation is attenuated by idiosyncrasies of the 4 pairs of raters allocated to that station, whereas in the mean rating across all stations, such idiosyncrasies can cancel out, increasing reliability and reducing attenuation. The internal consistency of the first impression rating was as on par with the internal consistency of the MMI performance rating. However, comparing both measures may appear unfair because the first impression rating had an advantage above the MMI performance rating: The same question was asked at every station, whereas the MMI performance rating was based on different questions, depending on the station’s tasks and rating instructions. But this objection is weak, because the mean MMI-rating across stations was supposed to reflect a single unidimensional construct—social competence, and therefore justifiably can be compared with another single unidimensional construct—first impression.

### The correlation of first impression with MMI performance

Correlations between first impression and subsequent MMI performance, assessed by a single rater, ranged between 0.21 and 0.38 when computed as mean correlations across 8 stations and between 0.28 and 0.46 when computed as correlations of mean scores across 8 stations. They were slightly lower when FIR and MMI ratings were made by different raters as compared to the same rater, for example r(FIR_1_, MMI_2_) = 0.37 and r(FIR_1_, MMI_1_) = 0.46, index 1 or 2 denoting rater identity (Table 2). This difference may reflect the effect of a desire to be consistent in the MMI rating after having put down a first impression which is possible only in the same-rater-condition but not in the different-raters-condition_._ However, the correlation between first impression and MMI persisted even when both variables were assessed by different raters. Therefore, the correlation of first impression with MMI performance cannot be simply taken as the effect of idiosyncratic biases of individual raters which shape their subsequent ratings in an effort for consistency. Such a correlation would disappear when ratings were not made by the same person. The correlation would only prevail if first impression was not completely idiosyncratic but to some extend uniform across raters, as suggested by the internal consistency of the FIR-scale. The moderate size of the correlation is in accordance with the effect sizes found in the meta-analysis of Ambady and Rosenthal (1992) about the accuracy of predictions for behavioral outcomes based on minimal information.

### First impression as predictor of a clinical examination OSCE

The validity criterion, two communication skill OSCEs assessed 18 months later in clinical examinations, was weakly predicted by a combination of first impression and MMI performance. Prediction did not depend on whether the rater who rated first impression was the same as the rater who rated MMI performance, or a different rater. The weakness of the prediction is no surprise since comparable correlations reported in the literature are small too—our study is no exception. The psychometric properties of OSCE-scores as obtained in clinical examinations are mostly unknown and variance is often small, thus low measurement quality diminishes predictive validity, moreover the selection for high MMI-scores has restricted its variance.

More surprising is that the MMI-performance rating did not contribute significant additional information after the first impression rating had entered the regression equation to predict psychosocial OSCE-performance.

### First impression as a proxy for an MMI?

If, after first impression is known, information about MMI-performance would not improve prediction of a validity criterion, how useful is it? In a more provocative way one could ask whether first impression could replace the MMI rating altogether. The correlation found between total scores for first impression and MMI-performance was r = 0.49. A correlation of this size cannot justify the replacement of one measure with another in most pragmatic contexts. For instance, body height correlating with body weight by about r = 0.5 is a bad proxy for body weight in most medical contexts. Still MMI-performance did not provide incremental validity above first impression in our study. As this finding might undermine the justification for MMI-methods developed with great effort in the last decades, it needs to be checked with methodological improvements, particularly a better psychometric quality of the validity criterion. The swiftness of first impression formation would explain to some extent why increasing the duration of a station’s MMI-task seems to have little impact on reliability (Reesa et al., 2016).

### Can we get rid of first impression?

Whenever humans assess other humans, first impression is involved. This is negligible when assessment does not depend on subjective evaluation as in objectively measurable athletic or cognitive performance, but it is hard to ignore in the assessment of psychosocial competence. If the validity criterion for a test of psychosocial competence based on a human observer again involves a human observer, first impression inevitably comes in on both sides, predictor and criterion. Thus, MMI and OSCE-results should be related simply because both assessments partly depend on first impression. Even if more realistic validity criteria can be employed, such as the evaluation of a physician’s social skills by patients or by para-medicals, first impression will always affect the measure. As self-presentation is part of personality, the ability to evoke first impressions may be viewed as a valid trait—after all, the impressions an applicant induces in others are likely to be similar in many of the applicant’s interactions, including those he or she will engage in as a medical doctor.

As has been shown in studies of first impression in zero-acquaintance-situations the manipulative component of narcissism would be part of such a trait (Back et al., 2010; Leckelt et al., 2015). To the extent that this is true, the MMI-procedure, by its correlation with first impression, would favor just those personalities it seeks to exclude. However, it has also been shown that narcissists do not fare well in more demanding interactional situations such as controversial discussions or dyadic decision making (Leckelt et al., 2015). MMI-stations may function this way, providing a correction for the otherwise deceptive allure of first impression. Still there are many unknowns: Is narcissism, as self-assessed by questionnaire, really a toxic trait? Do applicants for medical school who are charming at first sight will turn narcissistic exploiters in the long run? To what extend are raters able to detect and counteract narcissistic manipulation?

### Rater fatigue?

For many raters the task of evaluating a series of 48 short performances is probably engaging at first, then becomes routine and after a while burdensome (Christensen et al., 2018; Tavares & Eva, 2013). The cognitive load imposed by the task of judging with a fixed rating scheme may be increasingly avoided in favor of heuristic short cuts and snap decisions based on first impressions. If this is the case, the correlation of first impression with MMI performance rating should increase in the course of the rating session. A preliminary analysis of our data does not support this notion. However, as sequence-dependent effects are known in OSCE-like measures (Yeates et al., 2019), this question deserves investigation.

### An in-group casting new members?

In this study the wording for the first impression-rating did not match the MMI performance rating. A matching wording should have read: "Which level of MMI performance do you predict for this candidate?", but instead it was: "Does this applicant appear to be qualified for studying medicine?". In spite of this mismatch, the first impression rating moderately correlated with the MMI performance rating. Why? Perhaps the interview study by Christensen et al. (2018) hints at an explanation: in this study raters often testified to blending their specific rating task outlined in the instructions with the more general question known to them as the ultimate goal of the whole assessment procedure: could this candidate become a good medical doctor? In doing so they superposed the psychometric logic of measuring a psychological trait outlined by instructions with a different logic of their own: an in-group of medical professionals is choosing new members using tacit subjective cues for how well a candidate might fit the professional role. If this were the case and first impression rating would capture the same variance due to a tacit criterion of expected role-fit that is also hidden in the MMI-rating, then both variables would correlate and their contributions to the explained variance of a validity criterion would overlap. This is what we found. It suggests that an unintended and unadvertised in-group-casting-aspect is part of the MMI-rating and that this view influences the social competence assessment intended by the MMI-instructions and trainings. Medical professionals who are employed as raters probably lean to this view, and it is perhaps more fruitful to cultivate it instead of trying to suppress it. Consequently, the sampling of raters from the clinic’s staff which determines the mix of tacit criteria for expected role-fit becomes important and should be controlled carefully. Thus, the in-group-casting-view leads to a new appraisal of the sources of validity of MMI-like tests.

## Limitations

This study has preliminary character, it is more a proposition of design than a dependable empirical statement. Among its limitations, three are most noteworthy because they can be easily addressed in future studies:

1. The wording for the first impression-rating did not point directly to the impending MMI performance, but to a general qualification.
2. The instruction to explicitly record a first impression creates a mental awareness which is absent in the regular rater. Taking first impression from one rater and MMI performance rating from another, which we did, only addresses the repeated measurement aspect of the problem. Still, the instruction primes first impression  in both raters, whereas regular raters normally would not even think of the fact that they have first impressions. Only complete separation of the task of assessing first impression from the task of assessing MMI performance could avoid this source of confounding.
3. The validity criterion was based on only two communication skill OSCE-ratings. This can be improved by increasing the number of communication skill OSCE-ratings, and/ or by choosing other measures of social competence in a medical context.

In future studies first impression should be assessed by independent observers who are not MMI-raters. First impression ratings should focus on three tasks: (1) prediction of a person’s MMI-total score, (2) prediction of validity criteria such as success in communication skill OSCEs, and (3) assessment of physical attractiveness as a possible confounder. Furthermore the MMI may gain psychometric quality if it adopts the distinction between warmth, competence, and resilience which is prominent in the social psychological literature on social judgement (Breil et al., in press).

## Conclusion

First impression ratings generated a scale as internally consistent as the scale of MMI-ratings and both measures correlated with r = 0.49. They weakly predicted the mean of two psychosocial OSCEs obtained 18 months later with no incremental validity, neither for MMMI nor for first impression. As prediction did not depend on whether the MMI-rating was made by a rater who also rated first impression or by a different rater, first impression cannot be simply taken as the idiosyncratic bias of individual raters which shape their subsequent ratings in an effort for consistency. This would also be at odds with a multitude of findings in the social psychological literature. If first impression does influence subsequent ratings it would do so in a similar way for every rater, depending on the features presented by the applicant. On the other hand, first impression might just operate as the ability to predict outcomes, given sparse information about a person, but exert no influence on the rating process. To put it more bluntly: to what extent is first impression a bias that captures every rater equally (e.g. everyone deceived by narcissistic glamor), or a sober ability to predict outcomes? Perhaps future studies will be able to assess these alternatives.

## Data Availability

The datasets used during the current study are available from the corresponding author on reasonable request.

## Abbreviations

MMI: Multiple mini-interview
FIR: First impression
OSCE: Objective structured clinical examination
